# Risk–Benefit Assessment of Monomethylmercury and Omega-3 Fatty Acid Intake for Ringed Seal Consumption with Particular Emphasis on Vulnerable Populations in the Western Canadian Arctic

**DOI:** 10.3389/fnut.2017.00030

**Published:** 2017-07-26

**Authors:** Lena Gmelch, Holger Hintelmann, Brendan Hickie, Hermine Kienberger, Gary Stern, Michael Rychlik

**Affiliations:** ^1^Technical University of Munich, Freising, Germany; ^2^Water Quality Centre, Trent University, Peterborough, ON, Canada; ^3^Bavarian Biomolecular Mass Spectrometry Center, Technical University of Munich, Freising, Germany; ^4^Clayton H. Riddell Faculty of Environment, University of Manitoba, Winnipeg, MB, Canada; ^5^Queensland Alliance for Agriculture and Food Innovation, The University of Queensland, Coopers Plains, QLD, Australia

**Keywords:** methylmercury, omega-3 fatty acids, marine mammals, risk–benefit assessment, Inuit, ringed seals

## Abstract

Many northern Inuit communities rely on traditional food as major source of nourishment. An essential part of the traditional Arctic diet is marine mammals such as ringed seals or beluga. Being top predators, these animals are often highly contaminated with various toxins. In contrast, some tissues of marine mammals are also characterized by high amounts of n3-PUFAs (omega-3 polyunsaturated fatty acids). Here, we try to balance the risks associated with the consumption of different tissue types of ringed seals in terms of the neurotoxin monomethylmercury (MMHg) with the benefits of consumption due to high n3-PUFA concentrations. Fetuses are at the highest risk of neurological impairments because MMHg can easily cross the placental barrier. Therefore, women of childbearing age served as an indicator population for especially susceptible subpopulations. We calculated maximal weekly maternal portions sizes if mutual consumption of muscle and blubber tissue or liver and blubber tissue was assumed. Those weekly portion sizes resulted in an estimated overall IQ point gain of infants of 0, whereas the consumption of liver or muscle tissue without blubber could lead to an IQ loss. In contrast to former studies, our data do not generally prohibit the consumption of liver tissue. Instead, our results suggest that a maximal weekly consumption of 125 g liver tissue together with 1 g of blubber tissue is acceptable and does not lead to neurological damages in the long term. Similarly, the consumption of maximal 172 g muscle tissue can be balanced by the mutual consumption of 1 g blubber tissue.

## Introduction

Marine mammals such as ringed seals or beluga are top predators in the marine food web and mainly prey on fish and seafood. Consequently, they are highly contaminated with the neurotoxin monomethylmercury (MMHg) and other pollutants (1, 2). Since marine mammals are an important part of the traditional Arctic diet in northern Inuit communities, these contaminants may pose a certain risk for northern people (3–5). Many studies have found a significant relationship between fish consumption and Hg exposure. Resulting Hg body burdens in humans are strongly associated with the frequency of fish consumption and the concentration of MMHg in the consumed fish (6). MMHg is not only a potent neurotoxin that is effectively bioaccumulated through the aquatic food chain but there is now also sufficient evidence for its fetal neurotoxicity. It has been proven to cause neurological disabilities and irreparable damage to the central nervous system (2, 7–9). As MMHg can also cross the placenta barrier, fetuses are even at a higher risk due to the susceptibility of their developing brain and central nervous system (10). While clear dose–response relationships were not evident, many studies have now shown poor neurologic status and impaired development in newborns, infants, and children in response to *in utero* MMHg exposure [e.g., Ref. (11–13)], which has been compiled by the FAO/WHO to a correlation model of the mothers’ MMHg intake to the loss of IQ of their children during infancy (14). Exposure to high levels of inorganic Hg on the other hand has been shown in animal experiments to negatively affect kidney functions (14). However, marine food is also known for its high amount of long-chain polyunsaturated fatty acids (PUFA) (5, 15). Several studies have been conducted, demonstrating beneficial effects of an increased uptake of those essential fatty acids (FAs) (16–19). Uptake of omega-3 FAs was indicated to be linked with benefits to the brain and the visual system in developing infants (17, 20, 21).

Thus, several advisories for the consumption of fish and seafood have been made in terms of beneficial effects of n3-PUFA intake (22–26). Conversely, there are a large number of recommendations for reducing fish consumption due to risks of MMHg contamination (22, 27, 28). Both kinds of advisories may be contradictory and lead to confusion among consumers. Therefore, advisories aimed at balancing risks and benefits of the consumption of sea food should be issued to clarify statements and provide precise recommendations. Risk–benefit balancing of fish consumption is already a worldwide topic of growing interest (14, 24, 29, 30). In this study, we try to balance risks and benefits of the consumption of marine mammalian tissue focusing on the high-risk population “women of childbearing age.” Here, different tissue types of ringed seals from Ulukhaktok, NT, Canada, serve as an indicator species for the consumption of marine mammals.

## Materials and Methods

### Food Consumption Data

Food consumption data were gathered from the International Polar Year Inuit Health Survey (IHS) conducted in three Inuit jurisdictions in northern Canada [Inuvialuit Settlement Region (ISR), Nunavut, and, Nunatsiavut] in 2007 and 2008 (31). Data were obtained from 24-h dietary recall and semi-quantitative food frequency questionnaire (FFQ) over a period of 12 months among 2072 Inuit adults aged ≥18 years from 37 communities in the ISR (*n* = 6), Nunavut (*n* = 25), and Nunatsiavut (*n* = 6). 669 participants were women aged 18–40 years (childbearing age). Information regarding the consumption of traditional food was collected by applying a cross-sectional face-to-face administered FFQ as described in detail elsewhere (31, 32). Questionnaires were based on the Centre for Indigenous People’s Nutrition and Environment’s Inuit traditional FFQ, which were updated with IHS Steering Committees of ISR, Nunavut, and Nunatsiavut and adapted in that manner to reflect species available in each region.

### Sample Collection

Muscle, liver, and blubber tissue samples of 20 ringed seals (*Pusa hispida*) were obtained from the Department of Fisheries and Ocean (Winnipeg, MB, Canada). The archived samples were collected in Ulukhaktok, NT (formerly Holman Island, 70° 44′ 11″ N, 117° 46′ 5″ W) in the Western Canadian Arctic in the year 2012. All hunted animals were of mature age (6+ years). The process of sampling is described in detail elsewhere (33). Samples were stored frozen (−35°C), and subsamples were taken from the bulk sample after sufficient thawing. The thawed and homogenized subsamples were digested as required for the various analyses. Values were obtained in dry weight (d.w.) to account for possible loss of moisture during storage. To enable better comparison with previous data as well as food consumption advisories, measured d.w. data were converted into wet weight (w.w.) concentrations using typical water contents of 75, 70, and 5.5% for mammalian muscle, liver, and blubber tissue, respectively (34, 35).

### Materials and Reagents

All reagents and standards used for this study were at least of ultra-pure quality. Reagents were obtained commercially from the supplier given in parentheses: acetic acid (ACP Chemicals Inc., Montréal, PQ, Canada), chloroform (CHCl_3_), potassium hydroxide (Fisher Scientific Company, Ottawa, ON, Canada), Dogfish Muscle Tissue Reference Material DORM-4, Dogfish Liver Tissue Reference Material DOLT-5 (NRC, Ottawa, ON, Canada), isotope enriched monomethylmercurychloride standard was synthesized from isotope enriched Hg(0) (Trace Sciences International Corp. Richmond Hill, ON, Canada), milk fatty acid standard (QSE GmbH, Wolnzach, Germany), methanol (MeOH) (Fisher Scientific Company, Ottawa, ON, Canada), potassium chloride (BioShop Canada Inc., Burlington, ON, Canada), sodium acetate, sulfuric acid (Caledon Laboratory Ltd., Georgetown, ON, Canada), sodium chloride (NaCl) (J.T. Baker, Phillipsburg, NJ, USA), sodium ethyl borohydrate (NaBEt_4_), tributylphosphate (TBP), trinonanoylglycerol (TG-9:0) (Sigma-Aldrich Canada Co., Oakville, ON, Canada), *tert*-butyl-methylether (TBME) (Merck, Darmstadt, Germany), and trimethylsulfonium hydroxide (TMSH) (Macherey and Nagel, Düren, Germany).

### MMHg Levels

Concentrations of MMHg in all tissue samples were measured by isotope dilution inductively coupled plasma mass spectrometry (ICP-MS) interfaced to an automated Tekran 2700 Methyl Mercury Analyser (Tekran Instruments Corporation, Scarborough, ON, Canada). The exhaust vent of the Tekran 2700 was directly connected to a quadrupole ICP-MS (XSeries 2, Thermo Electron Corporation, Waltham, MA, USA). All samples were spiked with an enriched ${\text{CH}}_{3}^{202}\mathrm{HgCl}$ standard. Muscle and liver samples were additionally spiked with TBP to avoid foaming during distillation. Approximately 0.150 g of raw sample was distilled according to the procedure described elsewhere [140°C, Hg-free N_2_-gas stream, 60 mL/min flow rate (36)]. An aliquot of the distillate containing approximately 1,000 pg MMHg was diluted to 30 mL. Prior to analysis, samples were buffered to a pH of 4.5 by addition of 250 µL sodium acetate buffer (2 m). MMHg was ethylated *in situ* by adding 20 µL NaBEt_4_ (1%) to the sample vial that was immediately sealed. Derivatized MMHg was purged and preconcentrated on a Tenax trap. Subsequently, the formed ethylmethylmercury was introduced into the cold vapor-ICP-MS system after thermal desorption.

### n-PUFA Levels

Fatty acids were extracted using a modification of the Bligh and Dyer method (37). Approximately 2.2 g of muscle tissue were spiked with 100 µL TG-9:0 (0.1 g in 10 mL CHCl_3_) as internal standard (ISTD). Samples were homogenized with a mixture of 7.0 mL CHCl_3_/MeOH (1:1, v/v) using a tissue homogenizer (OMNI International, Kennesaw, GA, USA). The tip was washed in another 7.0 mL of CHCl_3_/MeOH (1:1, v/v) for another 30 s. After centrifugation of the combined solutions (7 min, 5,000 rpm, 5°C), the organic phase was transferred into a separating funnel. The extraction was repeated twice more to maximize recoveries. All supernatants were combined and extracted by addition of 21.0 mL of a NaCl solution (5.8%). Phase separation was allowed for 8 h or overnight. The chloroform layer was recovered, and the solvent evaporated in a rotary evaporator at 35°C under vacuum. Volumes used for the extraction of blubber tissue were adjusted to accommodate an initial sample weight of approximately 0.3 g. Thus, FAs were extracted using 3.0 mL of CHCl_3_/MeOH and 15.0 mL NaCl solution. Lipid extracts were dissolved in 500 µL TBME and mixed thoroughly. Of this solution, an aliquot of 100 µL was methylated with 50 µL TMSH and analyzed by gas chromatography with flame ionization detection (GC-FID) (Hewlett Packard 6890, Ramsey, MN, USA) as described in detail elsewhere (38). Milk fat of known FA composition was measured in parallel to identify peaks and determine response factors for quantification, using TG-9:0 as ISTD. For the milk fat, the same derivatization procedure was applied as described earlier.

The FA composition of liver tissue was not analyzed. The respective concentrations of omega-3 FAs were taken from data reported by Durnford and Shahidi (39). These data were obtained from ringed seals collected in the northeast areas of Newfoundland between April and June of 1995, 1996, and 1997.

### Quality Control

The quality assurance/quality control (QA/QC) protocol included the analysis of reagent, distillation, and method blanks. The Certified Reference Materials CRM DORM-4 and DOLT-5 were analyzed for MMHg, returning concentration which were 109 ± 4% (*n* = 3, ±1 SD) and 116 ± 10% (*n* = 13, ±1 SD) of the certified value for DORM-4 and DOLT-5, respectively. The detection limit was 1.2 ng g^−1^ (w.w.) for muscle tissue, 2.5 ng g^−1^ (w.w.) for liver tissue, and 0.3 ng g^−1^ (w.w.) for blubber tissue.

Reproducibility was evaluated by analyzing triplicates of each tissue type. Relative SDs for MMHg concentrations within triplicates were 1.8% for muscle, 10.2% for liver, and 17.0% for blubber tissue. The relative SD for EPA was 8.8% in muscle and 1.7% in blubber tissue and for DHA 14.5% in muscle and 3.0% in blubber tissue.

### Estimation of Dietary Exposure to MMHg, EPA, and DHA

Daily dietary exposure to MMHg, EPA, and DHA was obtained by combining consumption data (31) and the levels of MMHg, EPA, and DHA analyzed in this study. EPA and DHA concentrations in liver tissue were taken from data reported by Durnford and Shahidi (39).

### Risk and Benefit Assessment

The risks of the maternal consumption during pregnancy of several tissue types of ringed seals were calculated with respect to the effects on child IQ. Risks due to the MMHg concentration in marine mammalian tissue were balanced with benefits linked with n3-PUFA (omega-3 polyunsaturated fatty acids) intake. Equations were conducted as previously described by the Joint FAO/WHO expert consultation (14). Calculations for the risk based on the MMHg levels were as follows:

(1) IQ points gained=[MMHg]⋅s⋅(x7):64⋅9.3⋅(−0.18 or−0.7),

where [MMHg] is the MMHg concentration in µg g^−1^ w.w., *s* is the serving size in g, and *x* is the number of servings per week. 64 represents the estimated maternal weight in kg, 9.3 the correlation factor between maternal MMHg intake, and the maternal Hg level in µg g^−1^. The factors −0.18 and −0.7 account for the central and upper-bound estimate of IQ loss per μg g^−1^ in maternal hair, respectively.

Benefits of n3-PUFA intake were calculated with the following equation:

(2) $$\text{IQ points gained}=[\text{EPA}+\text{DHA}]\cdot 0.67\cdot s\cdot \left(\frac{x}{7}\right)\cdot 0.04,$$

where [EPA + DHA] is the concentration of EPA + DHA in mg g^−1^ w.w., 0.67 is the factor used to estimate DHA concentration from [EPA + DHA], and the factor 0.04 represents the gain of IQ points per mg of DHA intake per day. For a calculated IQ points gain greater than 5.8, values were corrected to the maximal value of 5.8 according to FAO/WHO (14).

Overall IQ gain was calculated per gram (*s* = 1 g, *x* = 1) by addition of the upper-bound estimate for MMHg concentration and the benefit of n3-PUFAs:

(3) IQ points gainedg={[MMHg]⋅1g7:64⋅9.3⋅(−0.70)}+{[EPA+DHA]⋅0.67⋅1g7⋅0.04}

## Results

For determining the daily intake of MMHg, EPA, and DHA associated with the consumption of different kinds of seal tissue, we analyzed muscle, liver, and blubber tissue for their MMHg concentrations and the concentration of several FAs (Table 1). MMHg values for liver tissue determined in this study (0.75 µg g^−1^ w.w.) were slightly higher than previous results (0.52 µg g^−1^ w.w.) from Dehn et al. (40), whereas concentrations of FAs were in comparable ranges. Total percentages of FAs were higher for muscle tissue in this study (2.83%) compared to results from the 90 s (1.85%, Table 1). Blubber, however, was lower in FAs (84.8% in 2012 compared to 93.6% in 1995–1997). Yet, the overall sum of EPA and DHA concentrations was the same in both studies with 0.87 mg g^−1^ in muscle tissue (w.w.). Blubber tissue was only somewhat lower concentrated (143 mg g^−1^ w.w.) in this study relative to previous findings (161 mg g^−1^ w.w.).

**Table 1:** Means ± SD (arithmetic) of the concentration of monomethylmercury (MMHg), percentages of fatty acids (FAs), concentrations of EPA, DHA, and the sum of EPA and DHA (EPA + DHA) of ringed seals from Ulukhaktok (formerly Holman Island, HO).

	MMHg [μg g^−1^]	FA [%]	EPA [mg g^−1^]	DHA [mg g^−1^]	EPA + DHA [mg g^−1^]
**HO 1995–97^a^**					
Muscle	n.a.	1.85 ± 0.53	0.43 ± 0.08	0.44 ± 0.02	0.87 ± 0.10
Liver	n.a.	3.71 ± 0.07	0.98 ± 0.02	0.71 ± 0.09	1.68 ± 0.11
Blubber	n.a.	93.55 ± 1.98	77.09 ± 9.37	83.54 ± 15.38	160.63 ± 24.75
**HO 2001^b^**					
Liver	0.52 ± 0.19	n.a.	n.a.	n.a.	n.a
**HO 2012^c^**					
Muscle	0.45 ± 0.27	2.83 ± 0.64	0.51 ± 0.11	0.37 ± 0.14	0.87 ± 0.22
Liver	0.75 ± 0.56	n.a.	n.a.	n.a.	n.a.
Blubber	0.02 ± 0.02	84.81 ± 6.94	55.71 ± 12.57	87.21 ± 14.84	142.92 ± 21.21

*All values reported are based on wet weight*.

*n.a. not analyzed, data depicted from ^a^Durnford and Shahidi (39), ^b^Dehn et al. (40), and ^c^this study*.

We used results analyzed in this study and concentrations of FAs in liver tissue determined by Durnford and Shahidi (39) to calculate the daily intake of MMHg, EPA, and DHA. Consumption data were obtained from IHS data (31) and applied for calculations of a median and maximal intake among consumers of seal tissue (Table 2). Daily intake of MMHg through the consumption of seal tissue was compared with the provisional tolerable weekly intake (PTWI) of 1.3 µg kg^−1^ body weight (bw) for all tissue types (30). We converted the PTWI into the respective daily intake and assumed a median bw of 64 kg for Canadian Inuit women of childbearing age (41). Thus, we calculated a tolerable daily MMHg intake of 12.1 µg. Median consumption of seal tissue did remain under the tolerable daily intake of MMHg for all tissue types (3.3, 1.7, and 0.04 µg from muscle, liver, and blubber tissue, respectively). However, a maximal consumption of seal muscle or liver exceeded the tolerable intake by the factor 3.2 for muscle and even 4.3 for liver tissue. In other words, either 2.2 maximal portions of muscle or 1.6 maximal portions of liver tissue per week were sufficient to exceed the PTWI (Table 3). Intake of n3-PUFAs was compared to the dietary reference value (DRV) of EPA + DHA of 350 mg (24). For the median intake scenario, only blubber tissue was found to be an important contributor for adequate daily intake of n3-PUFAs. A median intake resulted in an EPA + DHA uptake of 257 mg and therefore accounted for the advised daily consumption of 250 mg for the general population. Consequently, a maximal consumption of blubber tissue leads to an uptake of EPA and DHA exceeding the advisory of 350 mg for pregnant women by the factor of 18.8. Thus, a consumption of 9.5 median sized blubber portions or 0.4 maximal portions per week could help achieve the DRV. Muscle and liver tissue, however, were found to only be a supporting source of n3-PUFAs, even in the maximal consumption scenario (74 and 114 mg EPA + DHA for muscle and liver tissue, respectively). 33.0 and 21.4 mean portions would be necessary to achieve the DRV of EPA + DHA through consumption of muscle and liver tissue, respectively.

**Table 2:** Median and maximal daily intake of monomethylmercury (MMHg) and EPA and DHA (EPA + DHA) for women in Ulukhaktok among consumers of seal tissue (31).

	Median daily intake			Maximal daily intake		
	Portion size [g]	MMHg [μg]	EPA + DHA [mg]	Portion size [g]	MMHg [μg]	EPA + DHA [mg]
Muscle	7.3	3.28	6.38	85	38.2	74.3
Liver	2.3	1.71	3.87	68	50.6	114.4
Blubber	1.8	0.03	257.26	46	0.8	6574.4

**Table 3:** Portions of seal tissue to exceed the provisional tolerable weekly intake (PTWI) for the monomethylmercury intake and portions to achieve the dietary reference value (DRV) of EPA + DHA for median and maximal female consumers of childbearing age in Ulukhaktok.

	Median intake			Maximal intake		
	Portion size [g]	Portions to exceed PTWI	Portions to achieve DRV	Portion size [g]	Portions to exceed PTWI	Portions to achieve DRV
Muscle	7.3	25.4	383.9	85	2.2	33.0
Liver	2.3	48.6	633.4	68	1.6	21.4
Blubber	1.8	2545.0	9.5	46	101.8	0.4

We compared risks of consuming MMHg containing seal tissue with benefits resulting from the uptake of EPA and DHA. For the calculations, we applied the common metric “gain in IQ points” in infants due to maternal fish consumption as proposed by the FAO/WHO (14).

Table 4 summarizes the gain of IQ points per gram tissue for weekly consumption. To account for different approaches used to transfer data obtained in different studies, central and upper-bound estimate factors were applied (29), where the upper-bound estimate factor represents a more sensitive extrapolation. The central estimate factor, however, integrates results from the three main epidemiological studies (42). According to calculations of the FAO/WHO committee, we already assumed a risk when the consumption of fish lead to an IQ points loss in infants after applying the upper-bound estimate factor. Of all the tissue types analyzed, blubber was the sole tissue which was low enough in MMHg and high enough in EPA and DHA to result in an IQ points gain (0.5 points per gram blubber tissue). Muscle and liver tissue, however, were observed to lead to a loss in IQ points of −0.003 and −0.004, respectively. Considering mutual consumption of muscle and blubber tissue, 1 g of blubber tissue together with maximal 1,721 g of muscle tissue would balance out risks and benefits and result in a total IQ points gain of 0. For liver tissue, a mutual consumption of 1 g blubber tissue and maximal 125 g of liver tissue would result in a IQ points gain of 0.

**Table 4:** IQ gain per gram tissue of the consumption of several tissue types of ringed seals in Ulukhaktok due to monomethylmercury (MMHg) concentrations, eicosapentaenoic and docosahexaenoic acid concentrations (EPA + DHA), and after balancing MMHg, EPA, and DHA concentrations (total).

	IQ gain per gram tissue				
	MMHg		EPA + DHA	Total	
	Central estimate	Upper-bound estimate		Central estimate	Upper-bound estimate
Muscle	−0.002	−0.007	0.003	0.002	−0.003
Liver	−0.003	−0.011	0.006	0.004	−0.004
Blubber	0.000	0.000	0.547	0.547	0.547

*The central and upper-bound estimates refer to the different factors applied for calculations as done by the FAO/WHO 2011 (14)*.

## Discussion

We found that blubber tissue can be consumed by women of childbearing age in Ulukhaktok without any concerns regarding MMHg exposure due to the high amount of n3-PUFAs, which easily outweigh the risks from MMHg uptake. A gain of 0.5 IQ points per gram blubber tissue highlights the importance of this tissue type for the child’s neurodevelopment. Muscle and liver tissue, however, may not be consumed without potential negative impacts on the infant’s neurodevelopment, not even at minimal quantities (−0.003 and −0.004 IQ points per gram muscle and liver tissue, respectively). Consequently, even minimal consumption of those tissue types can show adverse effects on the infant’s neurodevelopment. However, the high percentage of n3-PUFAs in blubber can help counterbalance the risks of muscle and liver consumption, if those tissue types are consumed together. Accordingly, the weekly consumption of 1 g blubber tissue mitigates a consumption of as much as 172 g muscle tissue without any negative effect on the child’s IQ. This weekly consumption is equivalent to 2.0 maximal portions of muscle tissue. For liver tissue, 1 g of blubber tissue counterbalances the risk related to the consumption of 125 g, i.e., 1.5 maximal portions. On the basis of our study, we caution against the sole consumption of muscle or liver tissue for women of childbearing age in Ulukhaktok. We strongly recommend, however, mutual consumption of those tissue types with blubber tissue to better balance risks from MMHg uptake with the beneficial intake of high concentrations of n3-PUFAs. This evaluation is consistent with other reports highlighting the beneficial effects of PUFAs on infant development (43) and the ability of DHA to block out the negative effects of mercury exposure (44). Most of the insights regarding the benefits of PUFAs were gained from studies on fish consumption. However, other studies hypothesize that the body might assimilate the PUFA’s present in seal blubber more effectively than those from fish oil (45). This would suggest that our findings are in fact a conservative estimate for the benefits of PUFA in blubber.

Compared to fish consumption advisories from all over the world, seal tissue can be classified as “low contaminated” tissue with MMHg concentrations below 0.5 µg g^−1^ (14). However, fish is characterized by generally having much higher omega-3 FA concentrations. Consequently, for seal muscle and liver tissue, benefits for the neurodevelopment resulting from the intake n3-PUFAs cannot to the same extent counterbalance risks associated with MMHg uptake. The FAO/WHO consumption advisories for fish having less than 0.5 µg g^−1^ of MMHg is at least seven portions (1 portion = 100 g) a week (14). Consumption of fish with MMHg concentrations up to 1 µg g^−1^ and EPA + DHA concentrations >8 mg g^−1^ was advised to be limited to two servings per week. Regarding seal tissue, our results indicated that consumption should be limited to 172 g muscle or 125 g liver tissue, respectively, if those tissue types are consumed mutually with 1 g of blubber tissue. Those recommendations are in the same range as recently released fish consumption advisories of the FDA (46). Here, the committee advised consumers to limit weekly intake of white tuna to 6 ounces (equivalent of 170 g) and to avoid highly contaminated fish species such as tilefish from the Gulf of Mexico, shark, swordfish, or king mackerel. Low contaminated seafood such as shrimp, polluck, salmon, canned light tuna, tilapia, catfish, or cod were advised to be eaten at quantities of 8–12 ounces per week (equivalent of 227–340 g).

In summary, we would advise female Inuit of childbearing age from Ulukhaktok to confine their weekly consumption of marine mammalian tissue to 172 g ringed seal meat or 125 g ringed seal liver if consumed together with 1 g blubber tissue each. In other words, 1 tea spoon of blubber (approximately 4.5 g) can counterbalance the risks of the consumption of 9 mean portions of muscle tissue (770 g) or 8 mean portions of liver tissue (540 g). In contrast to previous recommendations, liver tissue must not necessarily be replaced with lower contaminated tissue types, as long as mutual blubber consumption is taken into account (31). Previous studies did not differentiate between burdens of total Hg and the much more neurotoxic MMHg, which represents only a small fraction of the total Hg in liver (on average only 3%). Once the species of mercury is taken into account, eating seal liver is less risky. Complete avoidance does not seem to be required and limited consumption is possible, especially if it is complemented with consuming blubber. However, one has to note that liver often contains high concentrations of inorganic mercury. Although inorganic mercury is not known to be a neurotoxin, it may be harmful for kidneys and cause nephrotic syndrome (8, 47). Moreover, some seal tissue, especially blubber, might additionally be contaminated with other toxins such as polychlorinated biphenols (PCBs), which are known to have adverse effects on the immune system (48, 49). Other authors also reported on neurodevelopmental risks associated with PCBs (50, 51). These studies found that children were vulnerable to prenatal PCB exposure, based on maternal body burden or cord serum PCB concentrations. However, we are not aware of studies, describing a quantitative relationship between the consumption of PCB contaminated food and resulting body burden in pregnant women or infants. Another study, for example, found that PCBs only had a relatively minor contribution to developmental endpoints (52). In a very recent and balanced evaluation of traditional Arctic foods consumed by women of childbearing age, Binnington et al. (53) concluded that blubber and meat from marine mammals is still essential for this population to maintain the intake of essential nutrients such as EPA and DHA. They recommend a diet consisting of “baseline traditional foods (TF),” which consist of 1.4 g seal blubber per day in a moderate intake scenario. According to the authors, this “specific consideration of baseline marine mammal TF intake rates, with potential replacement by fish TF products, can help ensure adequate nutrient intakes during reproduction while minimizing environmental pollutant exposures” of PCB and Hg. Our recommendation of 1 g seal blubber consumption per week is even lower thus further alleviating the risk from PCB intake. Consequently, the recommendations in this paper only consider and account for the neurotoxicological risks associated with MMHg, which is often the overriding concern for northern communities. Furthermore, such advice may need to be adjusted for different regions and/or subpopulations and for situations where fish or beluga are co-consumed with seal tissue during the same time period. Therefore, our study should not be seen as a general recommendation for Inuit all over the Arctic, but rather as a first reference point in balancing risks and benefits from the consumption of seal tissue with a focus on neurological risks. While the conclusions provided in this paper are based on the current situation, they may also serve as a starting point for projecting our recommendations into the future. First, the traditional diet of Inuits appears to change significantly due to environmental changes, which lead to a lower number of seals hunted in combination with lower seals’ fat and PUFA contents (54, 55). Second, along with the lower PUFA intake due to consumption of fewer seals, the general PUFA occurrence in marine mammals is also expected to decline due to global warming of sea water (56). Finally, our samples originate from 2012 and MMHg presumably increased since (57). Collectively, these developments point to the recommendation of further increasing the amount of blubber or other marine sources high in fat.

## Author Contributions

All authors participated in the conception of the research paper.

## Conflict of Interest Statement

The authors declare that the research was conducted in the absence of any commercial or financial relationships that could be construed as a potential conflict of interest.
